# Relevance of Kappa and Lambda Free Light Chains in Autoimmune Astrocytopathy Associated With Anti‐GFAP Antibodies

**DOI:** 10.1002/acn3.70103

**Published:** 2025-06-28

**Authors:** Michael Levraut, Romain Marignier, Mikael Cohen, Jeanne Benoit, Cassandre Landes‐Chateau, Elisabeth Maillart, Marion Cremoni, Barbara Seitz‐Polski, Anne‐Laurie Pinto, Pauline Dumez, Jérôme Honnorat, Christine Lebrun‐Frenay

**Affiliations:** ^1^ UR2CA‐URRIS, Unité de Recherche Clinique Côte d'Azur Université Nice Cote d'Azur, Centre Hospitalier Universitaire de Nice Nice France; ^2^ Laboratoire d'Immunologie Centre Hospitalier Universitaire de Nice Nice France; ^3^ Service de Neurologie, CRC‐SEP, and Centre de Référence des Maladies Inflammatoires Rares du Cerveau et de la Moelle Hôpital Neurologique Pierre Wertheimer Bron France; ^4^ Centre de Recherche en Neurosciences de Lyon, INSERM 1028 et CNRS UMR5292 Lyon France; ^5^ Université Claude Bernard Lyon 1 Lyon France; ^6^ Service de Neurologie, CRC‐SEP Centre Hospitalier Universitaire de Nice Nice France; ^7^ Service de Neurologie, CRC‐SEP, and Centre de Référence Des Maladies Inflammatoires Rares du Cerveau et de la Moelle Assistance Publique des Hôpitaux de Paris, Hôpital de la Pitié‐Salpêtrière Paris France; ^8^ Centre National de Référence Des Syndromes Neurologiques paranéoplasiques et encéphalites Auto‐Immunes Hospices Civils de Lyon Lyon France; ^9^ MeLiS, Équipe Synaptopathies et Autoanticorps (SynatAc) INSERM U1314 / UMR CNRS 5284 Lyon France

**Keywords:** autoimmune GFAP‐astrocytopathy, biomarkers, cerebrospinal fluid, kappa‐free light chains, lambda‐free light chains

## Abstract

**Introduction:**

The kappa‐free light chain (κ‐FLC) index is known to be highly sensitive and specific for diagnosing multiple sclerosis (MS), while little is understood about lambda (λ)‐FLC. This study assessed the κ‐FLC and λ‐FLC indices in autoimmune glial fibrillary acidic protein (GFAP) astrocytopathy.

**Methods:**

This multicenter study compares κ‐FLC and λ‐FLC indexes among patients with autoimmune GFAP astrocytopathy and sex‐ and age‐matched MS (positive control group) as well as symptomatic controls (headaches and small cerebral vessel disease, as the negative control group). We describe the correlation of both indexes with clinical variables and outcomes in the GFAP astrocytopathy cohort.

**Results:**

A total of 93 patients were included (31 in each group). The median κ‐FLC index was higher in the MS group (65.5 [35.7; 118.3]) compared to the GFAP astrocytopathy group (26.1 [11.4; 78.4], *p* = 0.062). With a κ‐FLC index threshold of 6.1, the proportion of patients with a positive κ‐FLC index was similar between the MS (94%) and GFAP‐astrocytopathy groups (84%, *p* = 0.425). The median λ‐FLC index was higher in the GFAP astrocytopathy group (45.5 [28.4; 96.9]) than in the MS group (10.6 [2.2; 29.1], *p* < 0.001).

In the GFAP‐astrocytopathy group, both CSF λ‐FLC and the λ‐FLC index at baseline were correlated with the last follow‐up mRS (*⍴* = 0.46, *r* ^2^ = 0.088, *p* = 0.014, and *⍴* = 0.32, *r* ^2^ = 0.12, *p* = 0.101, respectively).

**Conclusion:**

The κ‐FLC index alone cannot distinguish between autoimmune GFAP astrocytopathy and MS. We indicate a potential diagnostic and prognostic role of the λ‐FLC index in GFAP astrocytopathy that needs confirmation in independent cohorts.

## 1 Introduction

Intrathecal immunoglobulin (Ig) synthesis is prominent in numerous central nervous system (CNS) autoimmune disorders. Historically, it is proved within cerebrospinal fluid (CSF) analysis, showing a CSF‐restricted increase of total immunoglobulin‐G (IgG) or CSF‐restricted oligoclonal IgG bands (OCB) detection on isoelectric focusing, the latter being the gold standard [1]. The CSF quantification of kappa‐free light chains (κ‐FLC) has been more intensively studied for the past 10 years. It is reported to be at least as efficient as OCB in determining intrathecal Ig production [2, 3, 4], especially in multiple sclerosis (MS), with the advantage of being cost‐effective and fully automatized [5, 6, 7]. Many multicenter studies show that the κ‐FLC index, commonly used to estimate κ‐FLC intrathecal synthesis, can identify MS with reasonable accuracy [2, 3, 4, 8], with increased specificity for patients with high values of the κ‐FLC index [9, 10, 11]. In addition, quantifying the κ‐FLC index allows us to estimate clinical and MRI outcomes in MS, even in clinically and radiologically isolated syndromes [9, 10, 12, 13]. Together, these findings highlight why many authors recommend including κ‐FLC index metrics in the next revision of the MS diagnostic criteria. However, all studies have heterogeneous inflammatory control groups, potentially including patients with MS‐mimicking disorders such as neuromyelitis optica spectrum disorder (NMOSD) and myelin oligodendrocyte glycoprotein antibody‐associated disorder (MOGAD). Thus, the reported high specificity of the κ‐FLC index for MS diagnosis cannot be universally applied at the individual level in clinical practice. Currently, we lack data on both κ‐FLC and λ‐FLC indexes measured in CNS inflammatory spectrum disorders and their respective diagnostic and prognostic roles.

Autoimmune glial fibrillary acidic protein (GFAP) astrocytopathy is a recently identified disorder characterized by a phenotype of isolated meningoencephalitis or combined with myelitis or optic nerve involvement [14, 15, 16]. Its diagnosis relies on typical clinical and MRI presentations and CSF GFAP‐IgG identification by indirect immunofluorescence techniques and cell‐based assay (CBA) confirmation [14, 15]. Depending on clinical presentation, GFAP‐astrocytopathy can mimic many disorders, such as other autoimmune encephalitides, NMOSD, and MOGAD. It may sometimes present with optic nerve or focal spinal cord involvement and mimic MS [17, 18], especially since CSF analysis often shows intrathecal Ig synthesis [17, 18].

Given the reported high accuracy of the κ‐FLC index for diagnosing MS and the limited information on this biomarker in other CNS inflammatory disorders, we proposed measuring κ‐FLC and λ‐FLC in a cohort of patients with autoimmune GFAP‐astrocytopathy, alongside age‐ and sex‐matched MS patients (the positive control group) and age‐ and sex‐matched symptomatic controls (the negative control group). Additionally, we assessed the predictive value of κ‐FLC and λ‐FLC measures in patients with GFAP‐astrocytopathy.

## 2 Material and Methods

### 2.1 Standard Protocol Approvals, Registrations, and Patient Consent

The study was conducted following the Declaration of Helsinki and received approval and registration from the Institutional Review Board of the University Hospital of Nice Côte d'Azur (IRB number F20230425160909). All patients were provided with oral and written information, and non‐opposition to the research was obtained in accordance with French laws.

### 2.2 Study Participants

A series of patients diagnosed with autoimmune GFAP‐astrocytopathy tested positive for CSF GFAPα IgG between May 2017 and September 2023 at the Reference Center for Rare Brain and Spinal Cord Inflammatory Diseases (MIRCEM) and the French National Reference Center for Paraneoplastic Neurologic Syndromes in Lyon, France. Patients with sufficient stored blood and CSF samples for analysis were included in the GFAP‐astrocytopathy group. Both control groups (MS and SC) were randomly selected from the Nice MS tertiary center database (CyBIRD cohort, NCT 05056740). They were matched for age and sex to the autoimmune GFAP‐astrocytopathy cohort. All patients with MS met the 2017 McDonald criteria [19]. All patients in the SC group exhibited nonspecific neurological symptoms (i.e., headaches or psychiatric symptoms), normal brain MRI, and normal CSF analysis.

### 2.3 Collected Data

Data were collected for all participants, including age, sex, the time from symptom onset to serum and CSF sampling, and clinical symptoms at the time of sampling. The biological data gathered included CSF protein levels, CSF white blood cell count, and OCB status.

Other data, including tumor association, baseline modified Rankin Scale (mRS), treatment, and follow‐up information such as relapse and the last visit mRS, were gathered for GFAP‐astrocytopathy participants.

### 2.4 Blood and CSF Analysis

Cerebrospinal fluid GFAPα antibodies were measured using indirect immunofluorescent assays and confirmed with CBA at the National Reference Center for Neurologic Paraneoplastic Syndromes and Autoimmune Encephalitis, as previously reported [20].

OCB status was determined according to routine care in each center. IgG was separated from serum and CSF using isoelectric focusing on agarose gel. Bands were detected by immunoblotting or immunofixation.

The MS and SC groups were analyzed using fresh serum and CSF samples as part of routine care. Serum and CSF samples from the GFAP‐astrocytopathy participants were stored at −80°C in Neurobiotec, part of the Hospices Civils de Lyon biobank. Samples were frozen in dry ice at the immunology laboratory of Nice University Hospital. Upon receipt, samples were thawed and analyzed on the same day. Blood and CSF albumin, κ‐FLC, and λ‐FLC were measured by turbidimetry using the Optilite analyzer (The Binding Site, Birmingham, UK) and the Freelite Mx free light chain immunoassay (The Binding Site, Birmingham, UK), per the manufacturer's instructions. The determination of intrathecal synthesis of free light chains (FLC) was evaluated by calculating the FLC‐index using the formula: FLC‐index = (CSF FLC/serum FLC)/(CSF albumin/serum albumin). If the CSF FLC measurement was below the lower limit of detection of the analyzer (0.28 mg/L for CSF κ‐FLC and 0.74 mg/L for CSF λ‐FLC), a random value of 0.1 mg/L was assigned for calculating the FLC index. Kappa and λ‐FLC indexes were considered positive if (i) the CSF FLC concentration was detectable (above the lower limit of detection), with a κ‐FLC index ≥ 6.1 [11] or a λ‐FLC index ≥ 6.9 [3].

### 2.5 Statistical Analysis

Continuous variables were described using the median and interquartile range (first and third quartiles), while categorical variables were summarized by count and percentage. The associations between baseline covariates and κ‐FLC and λ‐FLC measures (CSF FLC values and FLC indexes) were analyzed nonparametrically using the Wilcoxon rank‐sum test for binary variables or the Kruskal–Wallis test for categorical variables with more than two levels. The correlation between baseline and last follow‐up modified Rankin scale (mRS) scores and κ‐FLC and λ‐FLC indexes was assessed using Spearman's coefficient.

*p* values less than 0.05 were considered statistically significant. Analyses were performed using R software, version 4.0.3, and figures made with the online application EasyMedStat version 3.35.

## 3 Results

### 3.1 Cohort Description

Serum and CSF samples were available for 93 patients: 31 in each group.

The main clinical phenotypes in the GFAP‐astrocytopathy group included meningoencephalitis (48%), meningoencephalomyelitis (35%), isolated encephalitis (10%), and isolated myelitis (7%), with one case of non‐longitudinally transverse myelitis affecting the cervical cord. Five patients (16%) in the GFAP‐astrocytopathy group presented with optic neuropathy related to optic disc edema. In the MS group, nine patients (29%) exhibited progressive disease at sampling. Cerebrospinal fluid analysis from the GFAP‐astrocytopathy group revealed higher CSF protein levels and white blood cell counts than the MS and SC groups (*p* < 0.001 for both comparisons). Oligoclonal bands in cerebrospinal fluid were found in similar proportions in both GFAP‐astrocytopathy and MS groups (85% and 84%, respectively, *p* = 0.999). All data are summarized in Table 1.

**TABLE 1:** Baseline characteristics of the groups.

	GFAP‐astrocytopathy	MS	Symptomatic control
	*n* = 31	*n* = 31	*n* = 31
Median age (IQR)	46 (39; 59)	47 (40; 58)	49 (38; 55)
Female, *n* (%)	17 (54.8)	17 (54.8)	15 (48.4)
Clinical phenotype			
GFAP‐astrocytopathy			
Meningoencephalomyelitis, *n* (%)	11 (35)	—	—
Meningoencephalitis, *n* (%)	15 (48)	—	—
Isolated encephalitis, *n* (%)	3 (10)	—	—
Isolated myelitis, *n* (%)	2 (6)	—	—
Optic disc involvement, *n* (%)	5 (16)	—	—
Peripheral neuropathy, *n* (%)	9 (29)	—	—
Multiple sclerosis:			
RR‐MS (%)		22 (71)	—
PP‐MS (%)	—	4 (13)	—
SP‐MS (%)	—	5 (16)	
Median mRS (IQR)	4 (3; 5)	—	—
Associated neoplasm, *n* (%)	6 (19)	0 (0)	0 (0)
Median CSF protein (g/L)	0.89 (0.61; 1.51)	0.40 (0.29; 0.47)	0.34 (0.28; 0.41)
Median CSF WBC (/mm^3^)	135 (68; 307)	3 (0; 5)	0 (0; 1)
OCB, *n* (%)	17/20 (85)	26 (84)	0 (0)
Median serum κ‐FLC (mg/L)	14.8 (10.7; 19.7)	12.8 (10.3; 17.0)	15.0 (11.7; 19.8)
Median CSF κ‐FLC (mg/L)	3.7 (1.6; 8.4)	3.6 (2.4; 8.5)	—
Patients with CSF κ‐FLC < 0.28 mg/L, *n* (%)	1 (3)	1 (3)	30 (97)
Median serum λ‐FLC (mg/L)	11.3 (10.3; 13.8)	12.6 (9.5; 14.8)	12.4 (10.6; 14.2)
Median CSF λ‐FLC (mg/L)	5.5 (3.2; 9.2)	1.0 (0.1; 1.8)	—
Patients with CSF λ‐FLC < 0.74 mg/L, *n* (%)	0 (0)	13 (42)	30 (97)

Abbreviations: κ‐FLC, kappa free light chains; λ‐FLC, lambda free light chains; CSF, cerebrospinal fluid; GFAP, glial fibrillary acidic protein; IQR, interquartile range; mRS, modified Rankin scale; MS, multiple sclerosis; OCB, oligoclonal bands; PP‐MS, primary‐progressive multiple sclerosis; RR‐MS, relapsing–remitting multiple sclerosis; SP‐MS, secondary‐progressive multiple sclerosis; WBC, white blood cells.

### 3.2 The κ‐FLC Index, Better Than the λ‐FLC Index, Was Associated With OCB Detection

**FIGURE 1:**
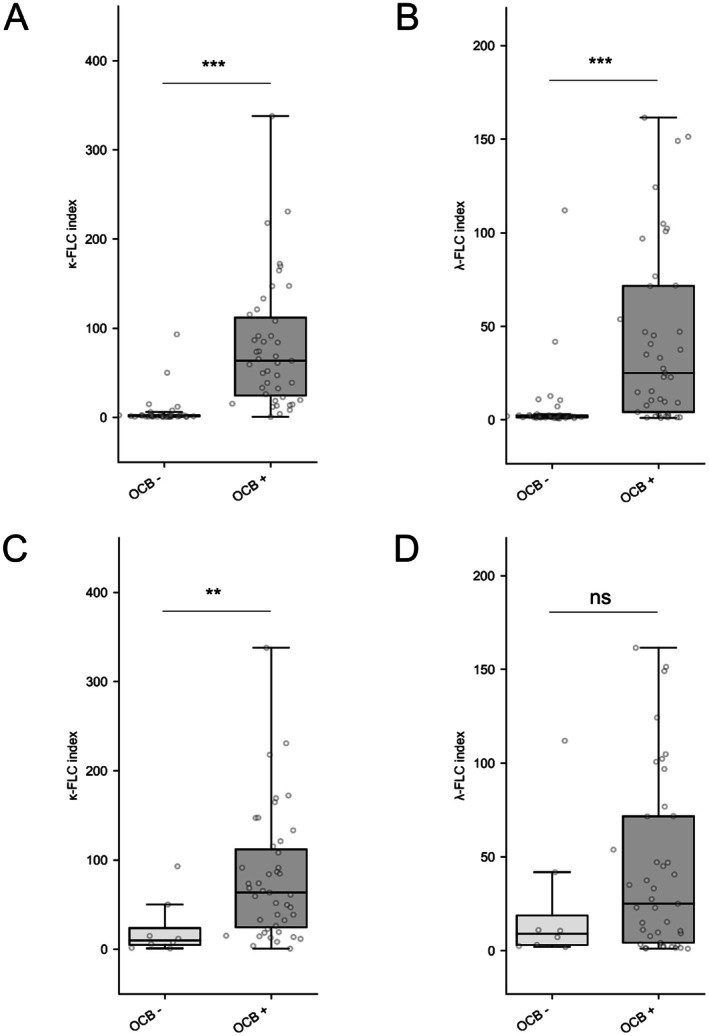
κ‐FLC and λ‐FLC associations with OCB. In all cohorts, the median κ‐FLC index (Figure 1A) and λ‐FLC index (Figure 1B) were significantly higher in OCB‐positive patients compared to OCB‐negative patients (*p* < 0.001 for both comparisons). ***Represents *p* value < 0.001. ** Represents *p* value <0.01. CSF, cerebrospinal fluid; FLC, free light chains; OCB, oligoclonal bands.

We first examined whether the κ‐FLC or λ‐FLC metrics were associated with OCB detection. In the all‐cohort evaluation, the κ‐FLC index and λ‐FLC index were higher in OCB‐positive (*n* = 43) than in OCB‐negative (*n* = 39) patients (median of 63.7 [24.5; 111.9] vs. 1.5 [1.1; 2.5], *p* < 0.001; Figure 1A for the κ‐FLC index—median of 26.0 [4.2; 71.6] vs. 1.9 [1.3; 2.4], *p* < 0.001; Figure 1B for the λ‐FLC index). Using the 6.1 threshold, the concordance between OCB and κ‐FLC index status was high (75/82, 91%) compared to the concordance between OCB and λ‐FLC index (6.9 threshold) status (64/82, 78%).

After excluding SC patients from the analysis (FLC not measurable in all but one patient), the median κ‐FLC index was higher in OCB‐positive (*n* = 43) than in OCB‐negative (*n* = 8) patients (63.7 [24.5; 111.9] vs. 9.9 [4.8; 23.7], *p* = 0.006, Figure 1C). However, the median λ‐FLC index was not statistically different between the positive and negative OCB subgroups (25.0 [4.2; 71.6] vs. 8.9 [2.9; 18.7], *p* = 0.298, Figure 1D).

### 3.3 κ‐FLC Metrics Were Elevated in Both GFAP‐Astrocytopathy and MS

**FIGURE 2:**
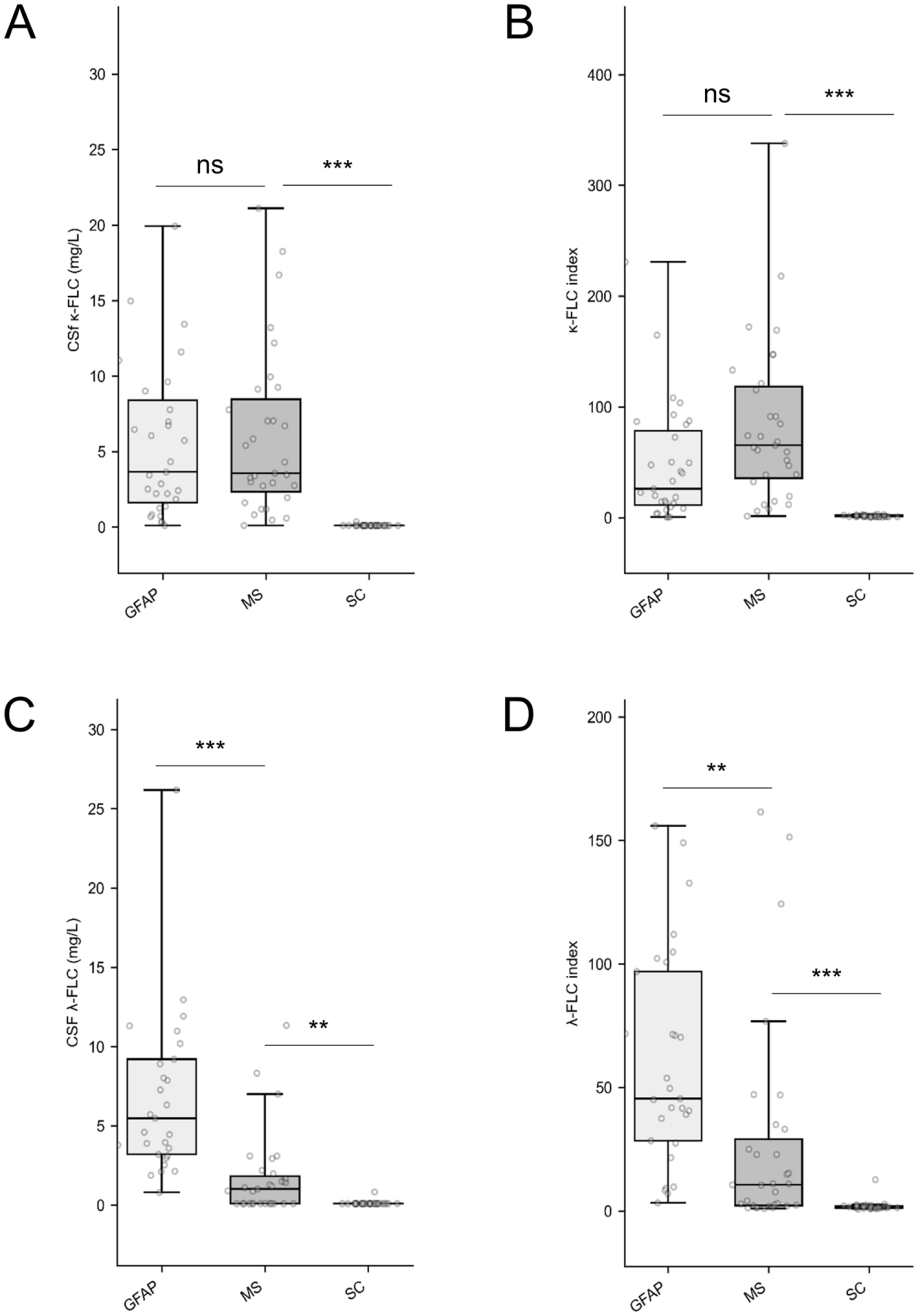
κ‐FLC and λ‐FLC biomarkers across groups. **Represents *p* value < 0.01. ***Represents *p* value < 0.001. CSF, cerebrospinal fluid; FLC, free light chains; GFAP, glial fibrillary acidic protein; MS, multiple sclerosis.

The median CSF κ‐FLC levels were comparable between patients in the GFAP‐astrocytopathy and MS groups (3.7 [1.6; 8.4] mg/L vs. 3.6 [2.4; 8.5] mg/L, *p* = 0.805). Only one patient in the SC group exhibited detectable but low CSF κ‐FLC (0.35 mg/L) (Figure 2A).

The median κ‐FLC index was lower in the GFAP‐astrocytopathy group than in the MS group (26.1 [11.4; 78.4] vs. 65.5 [35.7; 118.3], respectively; *p* = 0.062). The κ‐FLC index from the only SC patient with detectable CSF κ‐FLC was 3.1, which was considered negative (Figure 2B). When applying an independent cutoff of 6.1, the same proportion of patients with GFAP astrocytopathy (84%) and MS (94%) exhibited a positive κ‐FLC index (*p* = 0.425). All data regarding index positivity are provided in Table 2.

**TABLE 2:** Free light chains index positivity between GFAP astrocytopathy and MS.

	GFAP‐astrocytopathy	MS	OR (95% CI)	*p*
Positive κ‐FLC index	26 (84%)	29 (94%)	2.79 (0.50; 15.62)	0.425
Positive OCB + κ‐FLC index	15 (75%)	26 (84%)	2.43 (0.65; 9.01)	0.202
Positive λ‐FLC index	30 (97%)	18 (56%)	0.05 (0.01; 0.41)	< 0.001
Positive OCB + λ‐FLC index	16 (80%)	15 (48%)	0.3 (0.08; 1.14)	0.127

Abbreviations: κ‐FLC, kappa free light chains; λ‐FLC, lambda free light chains; IQR, interquartile range; OCB, oligoclonal bands.

### 3.4 λ‐FLC Metrics Were Higher in GFAP Astrocytopathy Than in MS

Median CSF λ‐FLC was higher in GFAP‐astrocytopathy (5.5 [3.2; 9.2] mg/L) compared to MS (1.0 [0.1; 1.8] mg/L, *p* < 0.001, Figure 2C). Similar results were observed after calculating FLC indexes, with the median λ‐FLC index being 45.5 [28.4; 96.9] in the GFAP astrocytopathy group and 10.6 [2.2; 29.1] in the MS group, respectively, *p* = 0.002 (Figure 2D). Using an independent cutoff of 6.9, nearly all patients in the GFAP‐astrocytopathy group (97%) exhibited a positive λ‐FLC index, while only 56% of patients with MS did (Table 2).

### 3.5 κ‐FLC and λ‐FLC Index Values Were Not Associated With a Specific Clinical Phenotype in Autoimmune GFAP‐Astrocytopathy

Since both κ‐FLC and λ‐FLC indexes were elevated in patients with autoimmune GFAP‐astrocytopathy and the clinical phenotype was heterogeneous, we examined a potential association between clinical presentation and FLC index values. There was no difference in the κ‐FLC and λ‐FLC index values between GFAP‐astrocytopathy patients with or without encephalitis, myelitis, cerebellar symptoms, optic nerve involvement, peripheral nerve involvement, or associated neoplasm (Table 3).

**TABLE 3:** Association of the kappa and lambda free light chains indexes with baseline clinical variables in the GFAP‐astrocytopathy group.

	Median κ‐FLC index (±IQR)		*p* Value	Median λ‐FLC index (±IQR)		*p*
	Yes	No		Yes	No	
Encephalitis	26.1 (±59.7)	47.7 (±93.1)	0.448	58.4 (±41.5)	71.5 (±58.4)	0.555
Cerebellar symptoms	48.7 (±63.5)	24.5 (±66.1)	0.367	63.5 (±50.8)	59.2 (±41.2)	0.809
Myelitis	19.9 (±35.0)	47.7 (±72.4)	0.241	51.1 (±45.2)	69.1 (±42.7)	0.288
Optic disc edema	18.6 (±71.1)	33.1 (±60.0)	0.872	40.7 (±27.5)	64.0 (±45.7)	0.335
Peripheral nerve involvement	19.9 (±37.2)	40.4 (±70.4)	0.856	41.8 (±23.9)	70.4 (±60.5)	0.403
Associated neoplasm	37.9 (±32.1)	28.0 (±75.5)	0.705	78.5 (±44.6)	55.9 (±43.6)	0.271

Abbreviations: κ‐FLC, kappa free light chains; λ‐FLC, lambda free light chains; CSF, cerebrospinal fluid; IQR, interquartile range.

### 3.6 High λ‐FLC Metrics Were Associated With Worse Neurological Outcomes in GFAP‐Astrocytopathy

**FIGURE 3:**
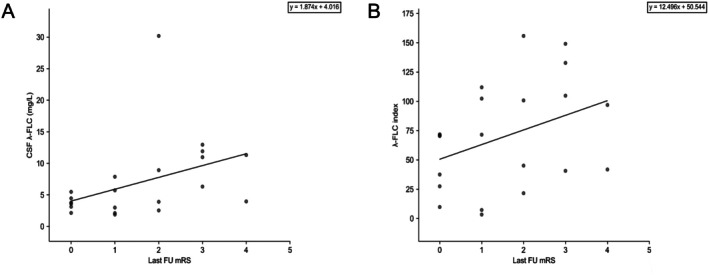
Association between last follow‐up clinical outcome and CSF λ‐FLC and λ‐FLC index in the GFAP‐astrocytopathy group. A low correlation was observed between CSF λ‐FLC (A) and λ‐FLC index (B) and the last follow‐up visit mRS score (*p* = 0.014 and *p* = 0.101, respectively). mRS, modified Rankin scale.

In the GFAP astrocytopathy group, κ‐FLC and λ‐FLC indices were not significantly associated with baseline mRS. Among the 21 patients with at least 6 months of follow‐up, both CSF λ‐FLC and λ‐FLC index correlated with the last follow‐up mRS (*⍴* = 0.56, *r* ^2^ = 0.16, *p* = 0.008 (Figure 3A) and *⍴* = 0.38, *r* ^2^ = 0.14, *p* = 0.092, (Figure 3B), respectively). Baseline CSF κ‐FLC and κ‐FLC index values were not associated with the last follow‐up mRS (*p* = 0.789 and *p* = 0.627, respectively; data not shown). Of the 25 patients whose treatment regimen was known, 22 (88%) received steroids, with additional immunosuppressive agents used as first‐line therapy in 7 (28%—rituximab, *n* = 5 and cyclophosphamide, *n* = 3, with one patient receiving both). Patients undergoing immunosuppressive therapy were more likely to have a favorable clinical outcome at the last follow‐up visit (median mRS of 1 [0; 2]) compared to those who did not (median mRS of 3 [2; 4], *p* = 0.037) and had higher CSF λ‐FLC levels (median of 7.9 [6.4; 11.2] vs. 3.8 [2.9; 7.0], *p* = 0.039) and λ‐FLC index (median of 53.8 [41.2; 104.5] vs. 43.4 [18.6; 71.6], *p* = 0.166) at baseline.

## 4 Discussion

Our study demonstrates that nearly all patients (84%) with autoimmune GFAP‐astrocytopathy exhibit κ‐FLC intrathecal synthesis and that the positivity of the κ‐FLC index alone cannot reliably distinguish patients with autoimmune GFAP‐astrocytopathy from age‐ and sex‐matched MS patients. A few individuals in the GFAP‐astrocytopathy group show very high κ‐FLC index values (> 100), suggesting that even elevated measures should be interpreted carefully before making an MS diagnosis. Intrathecal κ‐FLC synthesis has been observed in other conditions. Half of aquaporin‐4 (AQP4) positive NMOSD patients may show a slight but significant increase in the κ‐FLC index [21, 22], in contrast to a lower frequency of OCB positivity. Almost 70% of individuals living with HIV who present neurological symptoms have a positive κ‐FLC index [23]. These findings highlight the necessity for clinicians to consider clinical and MRI characteristics before assessing an MS diagnosis, even in patients with a high κ‐FLC index, to minimize misdiagnosis. Our results should be viewed with caution because our MS population is older (median age 47 years) with an over‐representation of the progressive MS phenotype. This approach was taken to best align patients with MS and GFAP astrocytopathy to reduce interpretation bias. While the MS phenotype does not influence the value of the κ‐FLC index [2, 11], it has been noted that CSF albumin concentration increases with age, associated with physiological changes in the blood–brain barrier [2] leading to a decreased κ‐FLC index [2, 24].

λ‐FLC is less studied than the κ‐FLC because its ability to diagnose MS or intrathecal immunoglobulin synthesis has been reported to be lower than that of the κ‐FLC index or OCB [2, 3, 25, 26]. λ‐FLC assessment in non‐MS inflammatory CNS disorders remains under‐researched, with the exception of Lyme neuroborreliosis (LNB) [27, 28]. Both studies indicated an increase in both κ‐FLC and λ‐FLC indices in LNB, with greater specificity derived from the λ‐FLC index [28]. Based on these studies and our findings, it seems that CSF λ‐FLC and the λ‐FLC index may assist in diagnosing non‐MS CNS inflammatory disorders. Our results also suggest that CSF λ‐FLC and λ‐FLC index may help identify GFAP‐astrocytopathy patients at risk for neurological disability. However, due to the insufficient data on treatment strategies and the limited power of the study, we did not perform multivariate analysis to determine whether λ‐FLC, treatment strategy, or both influenced clinical outcomes. We observe that patients undergoing immunosuppressive therapy exhibited higher λ‐FLC metrics at baseline. Nonetheless, better outcomes bolster our finding that λ‐FLC biomarkers correlate positively with clinical outcomes independently of treatment strategy. A focused study to evaluate whether λ‐FLC metrics could function as a biomarker for treatment strategy in autoimmune GFAP astrocytopathy would be worthwhile.

These results suggest that different CSF inflammatory profiles may exist for immune‐mediated CNS diseases. We observed a strong association between both OCB and positive κ‐FLC index, as reported in many studies. Additionally, we found a mild association between the λ‐FLC index and OCB positivity, with 78% of results being concordant. Therefore, λ‐FLC intrathecal synthesis may reflect a different process of CSF intrathecal immunoglobulin synthesis compared to that implicated in patients with κ‐FLC index positivity. As noted in MS, a low κ‐FLC/λ‐FLC ratio correlates with a progressive disease phenotype and higher disability, supporting the hypothesis of distinct B‐cell mediated inflammation in progressive MS that may be assessed by λ‐FLC biomarkers. At the same time, B‐cell Ig production has been reported in autoimmune GFAP‐astrocytopathy [14, 15, 29]. Anti‐GFAP antibodies targeting a cytosolic astrocyte antigen require disruption of the astrocyte membrane before binding their antigen [14, 15, 29]. According to human postmortem pathophysiological studies, CNS glial inflammation in GFAP astrocytopathy is primarily mediated by CD3 + CD8+ T cells, alongside the accumulation of C4d complement deposition [30]. Notably, CD20+ B cells cluster in the perivascular space and leptomeninges, accompanied by a moderate presence of CD79a plasmocytes [30], which could be involved in the meningeal Ig production observed in CSF analysis. However, the pathogenic role of anti‐GFAPα antibodies remains unresolved. Moreover, paraneoplastic or post‐viral autoimmunity has frequently been reported in GFAP‐astrocytopathy, emphasizing the role of T cells as the primary immune cells involved in CNS inflammation [14, 31]. Nevertheless, our findings suggest a pathogenic role for B cells, even though λ‐FLC metrics showed a weak correlation with clinical neurological outcomes. Thus, the mechanisms leading to B‐cell production of either CSF κ‐FLC or λ‐FLC may arise from T‐cell stimulation, such as the production of pro‐ or anti‐inflammatory cytokines, T‐cell antigen presentation, T‐helper cell activation, or T‐regulatory cell inhibition, as noted in MS [32, 33, 34, 35, 36]. Our findings raise numerous questions regarding B‐cell pathophysiology in both disorders.

Even if our results highlight the significance of κ‐FLC and λ‐FLC indexes in autoimmune GFAP‐astrocytopathy, our study has several limitations. First, while it is a rare and recently described disease, the number of included patients was relatively low, limiting our results' applicability to other cohorts. Future studies should aim to include a larger cohort to improve the generalizability of our findings. Second, although the clinical and biological data were collected independently at different centers (Hospices Civils de Lyon and Centre Hospitalier Universitaire de Nice, respectively), the study's retrospective nature may have introduced methodological bias. Our results should be validated in independent prospective studies. Third, the κ‐FLC and λ‐FLC metrics were obtained from frozen samples (for the GFAP‐astrocytopathy group) and fresh samples (for both the MS and SC groups), which could affect their comparison. However, it has been reported in independent cohorts that the nature of the samples does not influence κ‐FLC values [2]. Fourth, it is crucial to consider the incidence of MS and autoimmune GFAP‐astrocytopathy when interpreting a κ‐FLC index. Therefore, patients should only be screened for CSF GFAP‐IgG if they present with clinical and MRI red flags for MS to avoid unnecessary biological screening.

In conclusion, we demonstrated the high prevalence of positive κ‐FLC index in autoimmune GFAP‐astrocytopathy, which implies considering this diagnosis in patients with suspected CNS inflammatory disorder associated with κ‐FLC intrathecal synthesis and atypical features for MS. We also found that λ‐FLC metrics were elevated in almost all patients with autoimmune GFAP‐astrocytopathy, indicating a potentially different CSF inflammatory profile than those observed in MS and its potential diagnostic interest in such disorders. The prognostic role of λ‐FLC metrics and their pathophysiological role in autoimmune GFAP‐astrocytopathy are interesting and should be evaluated in dedicated studies.

## Author Contributions

Michael Levraut contributed to the study's design, data acquisition, analysis, and writing an essential part of the manuscript and figures. Romain Marignier contributed to the study's design, data acquisition, analysis, and reviewed the manuscript for intellectual content. Mikael Cohen contributed to the data acquisition, analysis, and reviewed the manuscript for intellectual content. Jeanne Benoit, Cassandre Landes‐Chateau, and Elisabeth Maillart contributed to the study's design and reviewed the manuscript for intellectual content. Marion Cremoni, Barbara Seitz‐Polski, Anne‐Laurie Pinto, and Pauline Dumez contributed to the data acquisition and review of the manuscript for intellectual content. Jérôme Honnorat and Christine Lebrun‐Frenay contributed to the study's design, data acquisition, analysis, and reviewed the manuscript for intellectual content.

## Conflicts of Interest

M. Levraut received travel compensation from BindingSite, the company that markets Optilite. R. Marignier received consulting fees from Alexion and UCB, honoraria for presentation or educational events by Alexion, Biogen, Amgen, Roche, and UCB, and support for attending meetings by Alexion. M. Cohen received consulting fees from Biogen, Sanofi, Janssen, Horizon Therapeutics, Merck, Celgene‐BMS, Alexion, and Ad Scientiam. J. Benoit has nothing to disclose related to this study. C. Landes‐Chateau has nothing to disclose related to this study. E. Maillart received research grants from ARSEP and Biogen and honoraria for presentations or educational events by Biogen, Janssen, Merck, Novartis, Roche, Sanofi, and Teva. M. Cremoni has nothing to disclose related to this study. B. Seitz‐Polski has nothing to disclose related to this study. A.L. Pinto has nothing to disclose related to this study. P. Dumez has nothing to disclose related to this study. J. Honnorat has nothing to disclose related to this study. C. Lebrun‐Frenay was invited as faculty by ECTRIMS or the European Charcot Foundation.

## Data Availability

The data supporting this study's findings are available at a reasonable request from the corresponding author.
